# Immune cell signatures and inflammatory mediators: unraveling their genetic impact on chronic kidney disease through Mendelian randomization

**DOI:** 10.1007/s10238-024-01341-z

**Published:** 2024-05-04

**Authors:** Yongzheng Hu, Fengyun Hao, Qian An, Wei Jiang

**Affiliations:** 1 Department of Nephrology, The Affiliated Hospital of Qingdao University, Qingdao, Shandong China; 2 Department of Pathology, The Affiliated Hospital of Qingdao University, Qingdao, Shandong China; 3 Department of Nephrology, Qingdao Central Hospital, Qingdao, Shandong China

**Keywords:** Chronic kidney disease, Mendelian randomization, Immune cell phenotypes, Inflammatory proteins, Genetic associations

## Abstract

Prior research has established associations between immune cells, inflammatory proteins, and chronic kidney disease (CKD). Our Mendelian randomization study aims to elucidate the genetic causal relationships among these factors and CKD. We applied Mendelian randomization using genetic variants associated with CKD from a large genome-wide association study (GWAS) and inflammatory markers from a comprehensive GWAS summary. The causal links between exposures (immune cell subtypes and inflammatory proteins) and CKD were primarily analyzed using the inverse variance-weighted, supplemented by sensitivity analyses, including MR-Egger, weighted median, weighted mode, and MR-PRESSO. Our analysis identified both absolute and relative counts of CD28 + CD45RA + CD8 + T cell (OR = 1.01; 95% CI = 1.01–1.02; *p* < 0.001, FDR = 0.018) (OR = 1.01; 95% CI = 1.00–1.01; *p* < 0.001, FDR = 0.002), CD28 on CD39 + CD8 + T cell(OR = 0.97; 95% CI = 0.96–0.99; *p* < 0.001, FDR = 0.006), CD16 on CD14–CD16 + monocyte (OR = 1.02; 95% CI = 1.01–1.03; *p* < 0.001, FDR = 0.004) and cytokines, such as IL-17A(OR = 1.11, 95% CI = 1.06–1.16, *p* < 0.001, FDR = 0.001), and LIF-R(OR = 1.06, 95% CI = 1.02–1.10, *p* = 0.005, FDR = 0.043) that are genetically predisposed to influence the risk of CKD. Moreover, the study discovered that CKD itself may causatively lead to alterations in certain proteins, including CST5(OR = 1.16, 95% CI = 1.09–1.24, *p* < 0.001, FDR = 0.001). No evidence of reverse causality was found for any single biomarker and CKD. This comprehensive MR investigation supports a genetic causal nexus between certain immune cell subtypes, inflammatory proteins, and CKD. These findings enhance the understanding of CKD's immunological underpinnings and open avenues for targeted treatments.

**Supplementary Information:**

The online version contains supplementary material available at 10.1007/s10238-024-01341-z.

## Background

Chronic kidney disease (CKD) represents a significant and escalating challenge to global health, with its prevalence and associated mortality rates surging upward [1]. The economic burden of CKD is particularly acute in low- to middle-income countries, where the costs associated with end-stage renal disease (ESRD) often exceed available resources. In these regions, over 50% of ESRD patients are at risk of discontinuing essential dialysis treatments due to the financial constraints, underscoring a critical need for more sustainable healthcare solutions [2]. Despite the growing impact of CKD on public health and economies worldwide, advancements in treatments that effectively slow renal function decline and avert the onset of ESRD are markedly lacking.

Inflammation serves as a central component in the development and progression of CKD, with a marked increase in pro-inflammatory cytokines like IL-6 and TNF-α contributing to renal damage, fibrosis, and a decline in kidney function [3]. This inflammatory state, present regardless of CKD's etiology, fosters glomerular and tubulointerstitial pathology, creating a harmful cycle where inflammation and oxidative stress perpetuate each other. The activation of pathways like NF-kB by oxidative stress further exacerbates inflammation, suggesting that inflammation is not only a consequence but also a potential initiator of CKD [4].

The genetic landscape of CKD points to an interplay between genetic predisposition and immune dysregulation, with immune system imbalances playing a crucial role in disease susceptibility and progression [5]. Treatments targeting immune modulation show promise, particularly when initiated early, in delaying the transition to end-stage renal disease. However, the direct causal link between immune cell dysfunction and CKD onset remains elusive, hindered by confounding factors in observational studies. There is a pressing need for a deeper investigation to unravel the complexities of the immune system's influence on CKD, which could unlock new avenues for therapeutic intervention and improve patient outcomes.

Mendelian randomization (MR) represents a formidable analytical tool, exploiting genetic variants to elucidate causal relationships between exposures and outcomes within observational data [6]. A seminal MR investigation recently illuminated the potential for inflammatory mediators as therapeutic targets in CKD, uncovering a causative linkage between heightened levels of C-reactive protein and the incidence of diabetic nephropathy [7]. MR's foundational principle—the random assortment of alleles during gametogenesis—effectively mitigates confounding factors and curtails reverse causation, thereby strengthening the validity of causal inferences drawn. The advent of bidirectional MR, an advanced iteration of the conventional approach, has proven pivotal in disentangling the complex bidirectional interplay inherent in biological systems, especially the reciprocal dynamics between exposures and outcomes [8].

In this comprehensive study, we leveraged publicly available data encompassing 731 immune cell subtypes and 91 immune-related proteins, alongside GWAS datasets pertinent to CKD. Our goal was to dissect and illuminate the complex interactions and causal relationships within this framework. Utilizing a bidirectional Mendelian randomization analysis, we sought to determine not only the influence of immune cell subtypes and cytokines on CKD risk but also the potential feedback loop wherein CKD may alter immune profiles. This dual approach provided a robust assessment of the reciprocal causality between systemic immune regulation and renal function dynamics. The integration of extensive immune cell subtype information with inflammatory protein data presents a nuanced view of the genetic underpinnings that could drive CKD pathogenesis and progression, offering a rich source of insights for identifying novel therapeutic avenues.

## Methods

### Study design

**Fig. 1:**
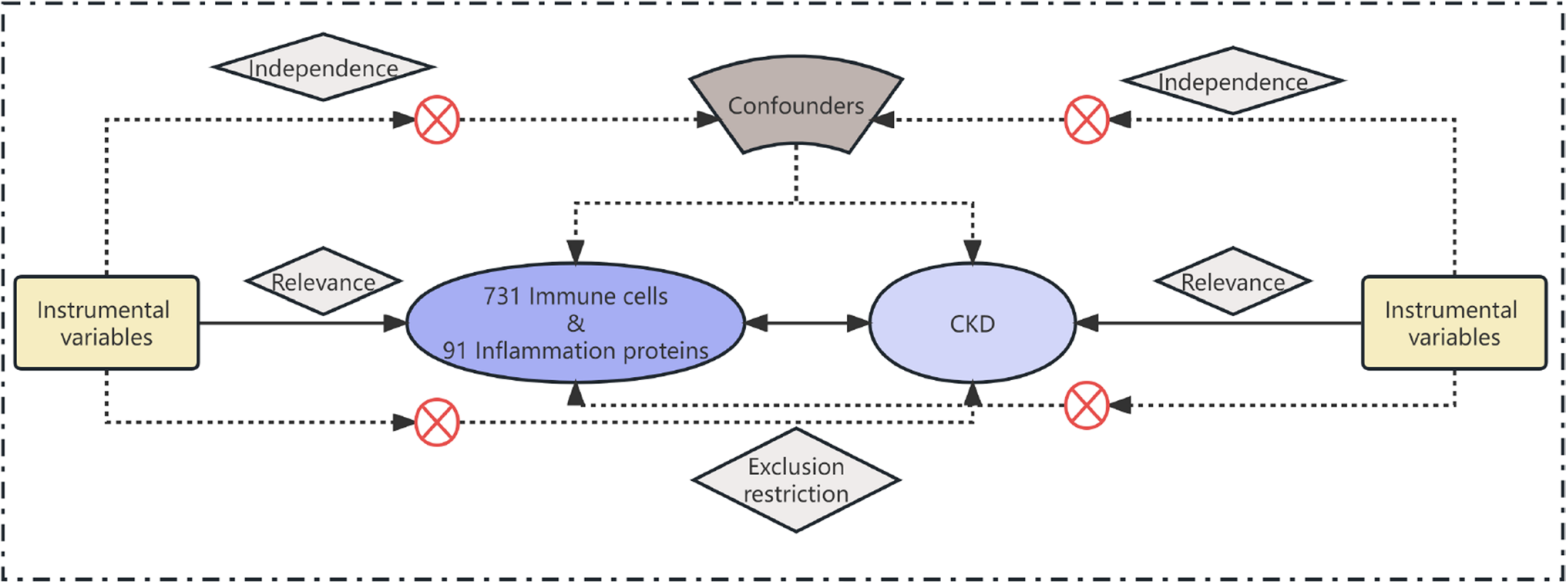
Schematic of the study design in the bidirectional Mendelian randomization analysis. Significant instrumental variables were selected for 731 immune cell subtypes, 91 inflammatory proteins and CKD, and the bidirectional causalities were then explored. Assumptions of a Mendelian randomization analysis were illustrated. Broken points represent potential pleiotropic or direct causal effects between variables that would violate Mendelian randomization assumptions. *CKD* chronic kidney disease

In our study, we deployed a two-sample MR approach to investigate the causal impact of 731 immune cell subtypes and 91 inflammation-related proteins on CKD. Our analysis adhered to the stringent MR assumptions of relevance (genetic variants must be associated with the exposure), independence (variants must be uncorrelated with confounders), and exclusion restriction (variants influence the outcome solely through the exposure) [9]. Utilizing GWAS summary statistics, we selected genetically significant SNPs that underpin the immune cell subtypes and inflammatory proteins involved in CKD. The overall design is shown in Fig. 1. More information about immune cell subtypes and inflammation-related proteins can be found in Tables S1 & S2.

### Ethical approval

In conducting this study, we utilized publicly accessible GWAS summary statistics, thereby circumventing the need for individual-level data acquisition. Ethical clearance for the original data collection was duly obtained by each contributing study's institutional review board, where all participants provided informed consent. This study has been registered and approved by the ethics review board of ethics committee approval of the affiliated hospital of Qingdao University. Ethics approval number: QYFY WZLL 28270.

### Exposure and outcome data sources

To estimate the effects of SNPs on a diverse array of immune cell subtypes, including various T cell classifications (naïve, CM, EM, TD, Treg, and NKT) as well as B cell subsets (naïve, unswitched memory, switched memory, and transitional), we utilized GWAS summary statistics from Orru et al. [10]. This analysis incorporated data from 3,757 individuals, assessed by flow cytometry within a Sardinian founder population. The GWAS Catalog provides comprehensive access to these data (accession numbers GCST0001391 to GCST0002121), covering 731 immune subtypes spanning absolute and relative cell counts, median fluorescence intensities indicative of surface antigen presence, and morphological characteristics. For the evaluation of SNPs linked to circulating inflammatory proteins, encompassing a panel of 91 proteins, we referred to GWAS summary statistics (accession numbers GCST90274758 to GCST90274848) provided by Zhao et al., which synthesized findings from 11 cohorts of European ancestry, totaling 14,824 participants [11]. Finally, the potential influence of these genetic variants on CKD risk was scrutinized using data from the CKDGen consortium's fourth round of analyses [12]. This encompassed 41,395 CKD cases and 439,303 controls, all of European descent, with rigorous quality control and imputation measures applied. For adults, GFR was estimated using the CKD-EPI equation, while for younger individuals, the Schwartz formula was applied, with eGFR readings adjusted to a range between 15 and 200 ml/min/1.73 m^2^. CKD was clinically defined by an eGFR threshold under 60 ml/min/1.73 m^2^, aligning with established diagnostic criteria.

### Instrumental variable selection

In defining the genetic underpinnings of immune cell signatures and inflammation-related proteins, our study adopted a genome-wide significance threshold of *p* < 1 × 10^–5^ to identify strongly associated SNPs, while a more stringent threshold of *p* < 5 × 10^–8^ was reserved for associations with CKD. We utilized the clumping functionality of PLINK software (version v1.90) to ensure the independence of our instrumental variables (IVs) [13], setting a linkage disequilibrium (LD) r2 threshold of < 0.1 within a 1000 kb span, using the 1000 Genomes Project as a reference. The explained variance in exposure by each SNP was quantified by the R2 value, and the strength of the instruments was gauged using the F-statistic, with an F > 10 indicating robust instruments [14]. To adhere to the independence assumption critical to MR analysis, we searched the PhenoScanner V2 to find out SNPs showing suggestive association (*p* < 10^−5^) with risk factors and excluded SNPs with any suggestive pleiotropic effects on CKD, such as those with associations with hypertension or diabetes [15]. To preserve the analytical integrity, we aligned the effect estimates by harmonizing the SNPs associated with both exposure and outcome, ensuring concordance with the same effect allele. SNPs with palindromic sequences and intermediate allele frequencies, or those exhibiting incompatible alleles, were systematically excluded [16].

### Statistical analyses

For our primary analysis, we employed the inverse variance-weighted (IVW) methods to estimate the exposure's impact on the outcome, assuming all MR prerequisites are met [17]. The use of random effects IVW was prioritized over fixed effects in cases where the null hypothesis was refuted [18]. Recognizing that the IVW method presupposes all genetic variants as valid instrumental variables—a condition potentially unmet in reality—we also incorporated weighted median (WM) approaches, which are less dependent on this assumption, to yield consistent causal estimates [19]. To enhance the robustness of our findings, we performed sensitivity analyses, including the weighted mode analysis, and the MR-Egger approach [20], the latter providing causative effect estimations under the Instrument Strength Independent of Direct Effect (InSIDE) assumption. Aware of the inherent risks of false positives in multi-dataset analyses, we applied a Bonferroni-corrected significance threshold for an added layer of stringency. An exposure's causal effect was deemed indicative with a nominally significant false discovery rate (FDR < 0.05) in the IVW method, bolstered by consistent results across sensitivity tests. The heterogeneity of instrumental variables was scrutinized using Cochran’s Q test, with significant heterogeneity marked by *Q*_*p* < 0.05 [21]. Further, the MR pleiotropy residual sum and outlier (MR-PRESSO) test was utilized to identify and adjust for horizontal pleiotropic outliers, refining our effect estimations [22]. Directional pleiotropy was also examined via the MR-Egger regression intercept, with significance set at *p* < 0.05. The leave-one-out (LOO) analysis and graphical assessments through funnel and scatter plots provided additional scrutiny of the robustness and symmetry of the effect estimates [23]. Our bidirectional MR analyses, considering the potential reciprocal effects between CKD and immune-related markers, employed SNPs associated with CKD as instrumental variables. These comprehensive analyses were executed using the TwoSampleMR (version 0.5.7) and MR-PRESSO (1.0) packages in R (4.2.3), with forest plots generated via the Forestplot package (1.1.1), ensuring a meticulous and transparent presentation of our results.

## Result

### Selection of instrumental variables

In our analysis, we evaluated 731 immune cell phenotypes and 91 inflammatory proteins, identifying genetic variants as instrumental variables with robust associations (F-statistics ranging from 20 to 282). For the reverse Mendelian randomization, genetic variants pertinent to CKD were scrutinized as potential instrumental variables. Post-exclusion of any potential pleiotropy, a recalculated F-statistic affirmed the strong association of 22 SNPs with CKD, each exhibiting an F-statistic of 62, indicating substantial relevance to the exposure factor. The details of these SNPs are shown in Table S3.

### Causal link between immune cells and CKD

**Fig. 2:**
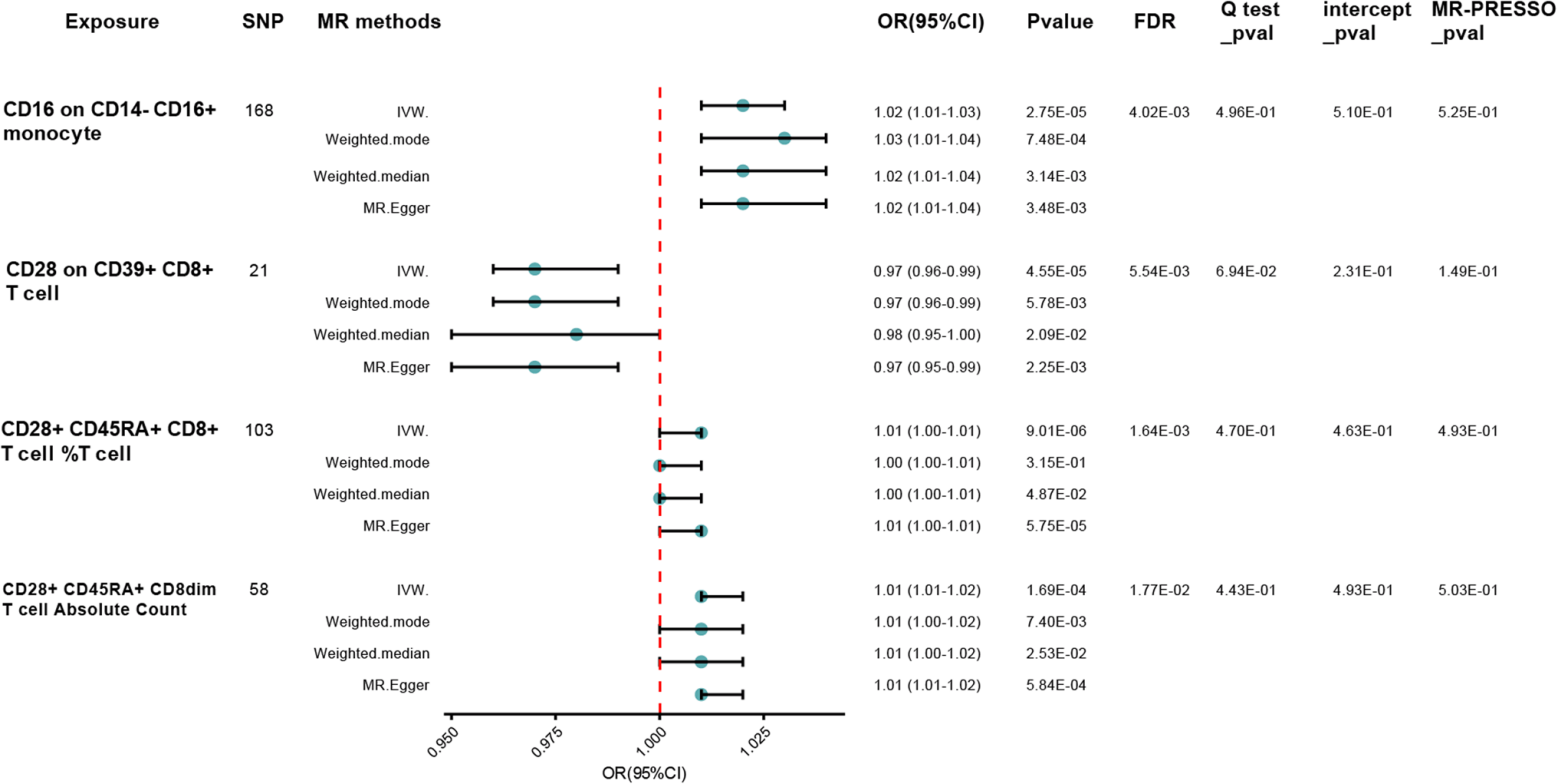
Causal correlations of 731 immune cell subtypes on CKD %Tcell: relative count of T cell; *SNP* single nucleotide polymorphisms; *IVW* inverse variance-weighted; *OR* odd ratios; *CI* confidence interval; *FDR* false discovery rate

**Fig. 3:**
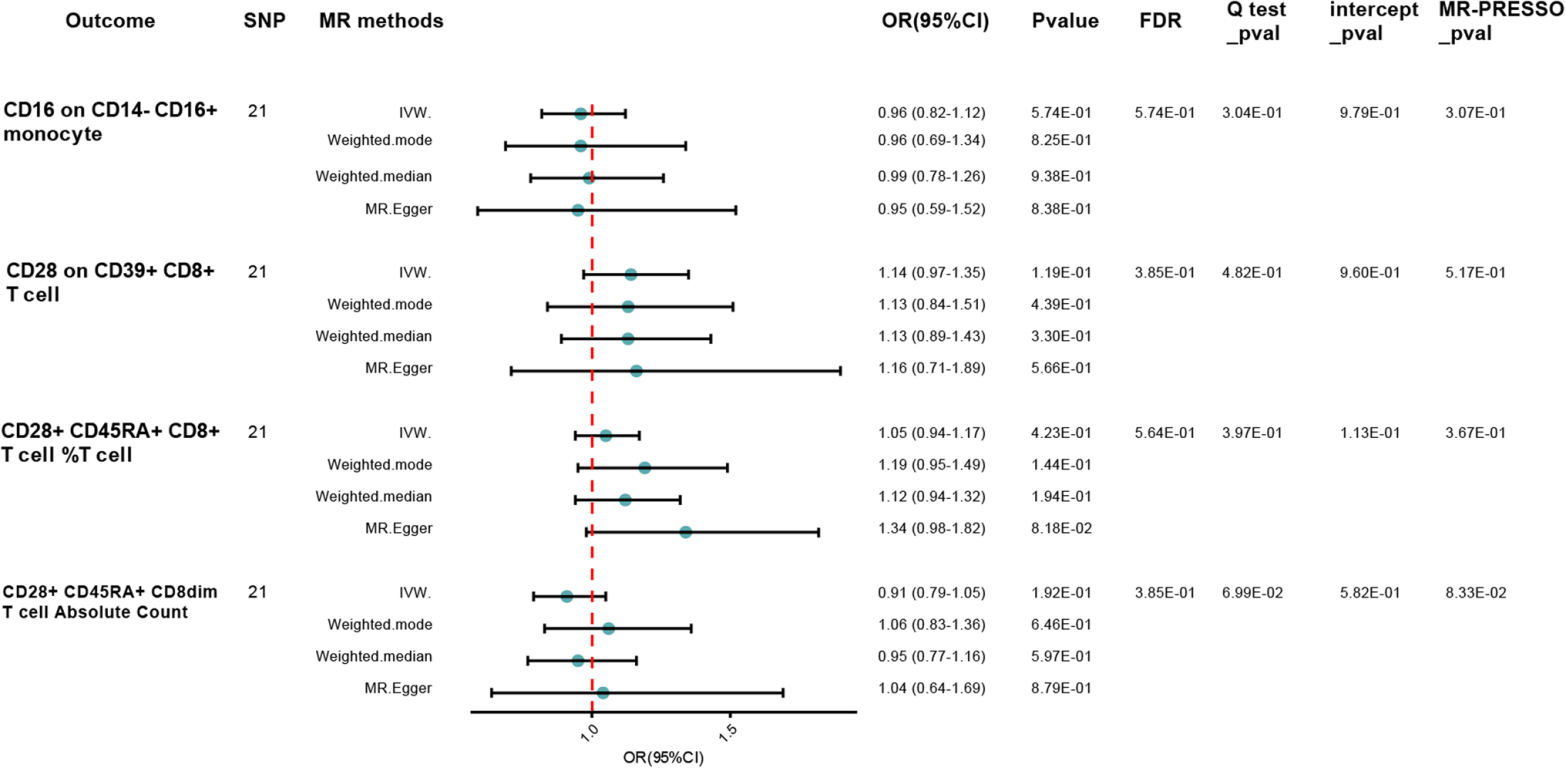
Causal correlations of CKD on selected immune cell subtypes %Tcell: relative count of T cell; *SNP* single nucleotide polymorphisms; *IVW* inverse variance-weighted; *OR* odd ratios; *CI* confidence interval; *FDR* false discovery rate

We observed that higher absolute count of CD28 + CD45RA + CD8dim T cell was robustly associated with increased CKD susceptibility using IVW method [odds ratio (OR), 1.01; 95% confidence interval (CI), 1.01–1.02; *p* < 0.001, false discovery rate (FDR), 0.018]. We also found suggestive evidence that relative count of CD28 + CD45RA + CD8 + T cell was positively associated with disease risk (OR = 1.01; 95% CI = 1.00–1.01; *p* < 0.001, FDR = 0.002). We next extended our analyses by further measuring the causal estimates of median fluorescence intensities (MFIs) of immune cells on CKD risk. The IVW method revealed that an increase of CD28 on CD39 + CD8 + T cell was associated with a lower risk of CKD (OR = 0.97; 95% CI = 0.96–0.99; *p* < 0.001, FDR = 0.006), which was supported by other MR methods. The results indicated that CD16 on CD14- CD16 + monocyte (OR = 1.02; 95% CI = 1.01–1.03; *p* < 0.001, FDR = 0.004) subset was causally associated with CKD. For the remaining cellular subtypes, our analyses indicated no significant causal effects on the risk of chronic kidney disease, with estimates attenuating toward null. The full analysis results of MR can be viewed in Table S4. Rigorous sensitivity analyses reinforced the robustness of our results and revealed no evidence of bias stemming from genetic pleiotropy. Leave-one-out procedures further substantiated the absence of undue influence by individual SNPs on our effect estimates (Fig. S1). Funnel plots (Fig. S2), upon visual examination, did not unveil any detectable directional pleiotropy, a finding corroborated by the nonsignificant MR-Egger regression intercept (Fig. S3). Additionally, the Cochran Q test did not signal any significant heterogeneity across the instrumental variables. We then perform a reverse Mendelian randomization analysis, and the analysis results are saved in Table S5. In addition, genetic predisposition to CKD as exposure did not have causal impact on the absolute counts of CD28 + CD45RA + CD8dim T cell(OR = 0.91; 95% CI = 0.79–1.05; *p* = 0.192, FDR = 0.385), relative count of CD28 + CD45RA + CD8 + T cell (OR = 1.05; 95% CI = 0.94–1.17; *p* = 0.423, FDR = 0.564), CD28 on CD39 + CD8 + T cell(OR = 1.14; 95% CI = 0.97–1.35; *p* = 0.119, FDR = 0.385), or CD16 on CD14- CD16 + monocyte (OR = 0.96; 95% CI = 0.82–1.12; *p* = 0.574, FDR = 0.574) in bi-directional MR analyses. The forward and reverse causal estimates between immune cell subtypes and CKD are as summarized graphically in Figs. 2 and 3, respectively.

### Causal link between inflammatory proteins and CKD

**Fig. 4:**
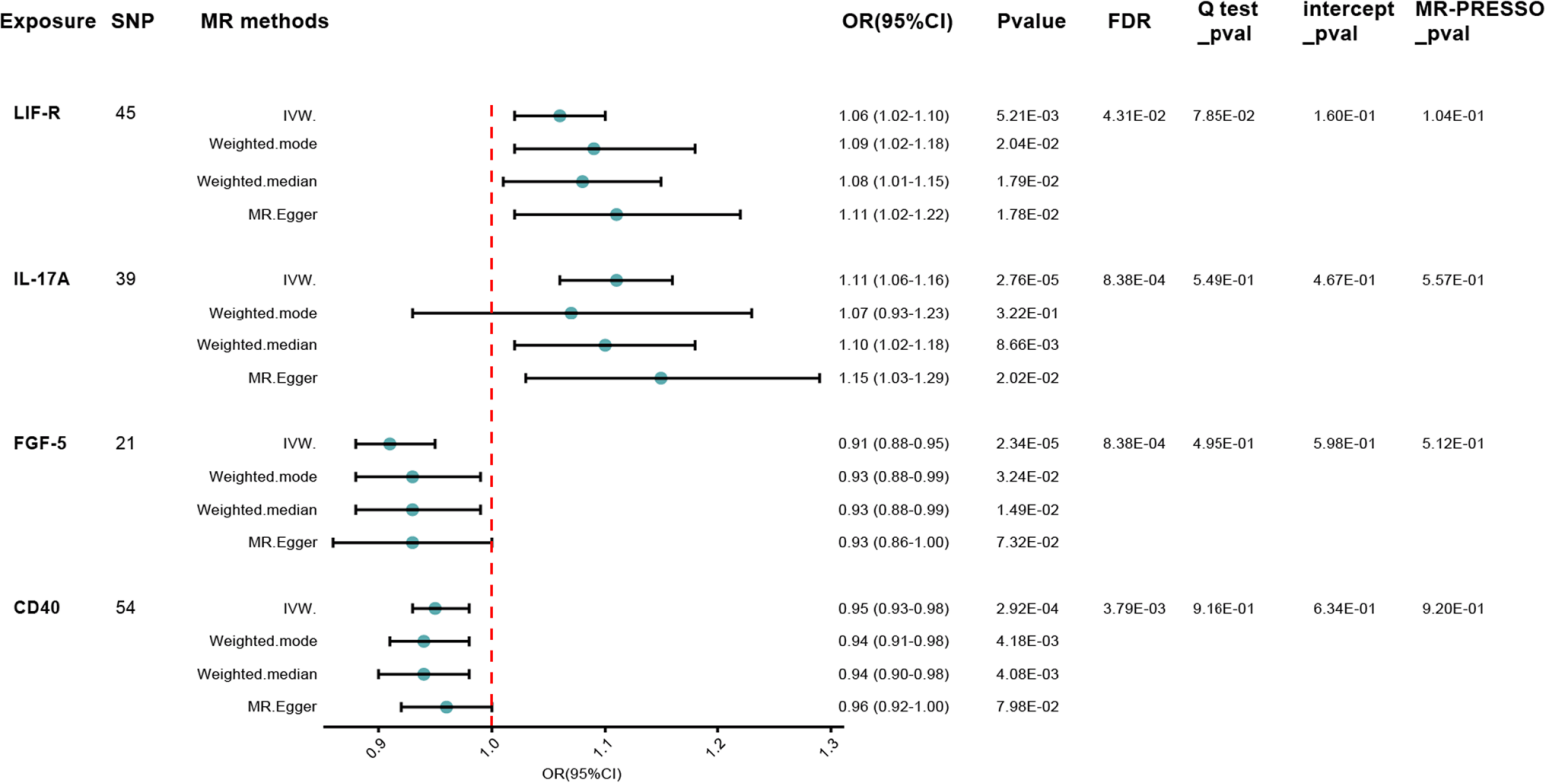
Causal correlations of 91 inflammatory proteins on CKD LIF-R: leukemia inhibitory factor receptor; *IL-17A* interleukin-17C; *FGF-5* fibroblast growth factor 5; *CD40* CD40L receptor; *SNP* single nucleotide polymorphisms; *IVW* inverse variance-weighted; *OR* odd ratios; *CI* confidence interval; *FDR* false discovery rate

**Fig. 5:**
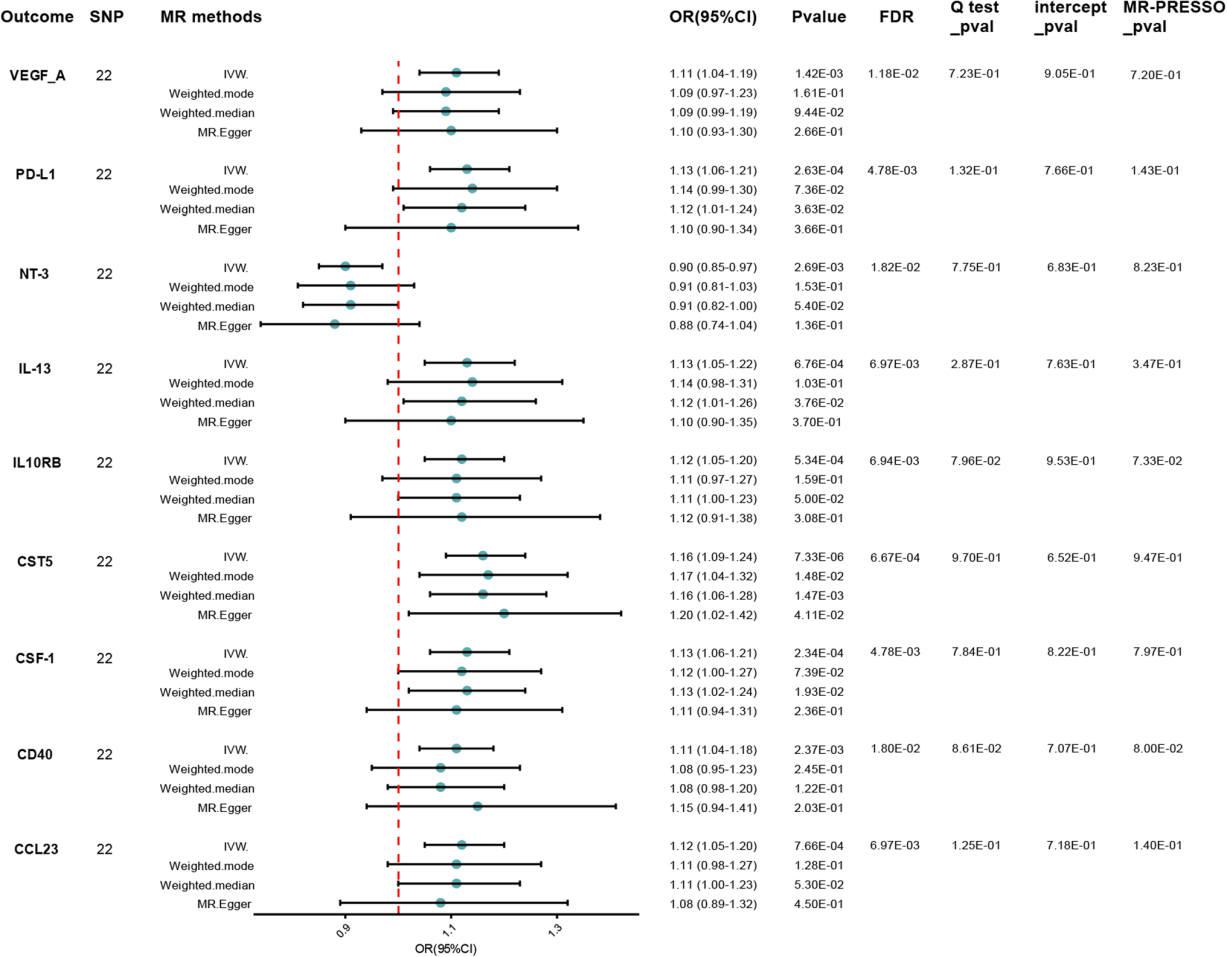
Causal correlations of CKD on 91 inflammatory proteins *VEGF_A* vascular endothelial growth factor A; *PD-L1* programmed cell death 1 ligand 1; *NT-3* neurotrophin-3; *IL-13* interleukin-15 receptor subunit alpha; *IL10RB* interleukin-15 receptor subunit alpha; *CST5* cystatin D; *CSF-1* macrophage colony-stimulating factor 1; *CD40* CD40L receptor; *CCL23* C–C motif chemokine 23; *SNP* single nucleotide polymorphisms; *IVW* inverse variance-weighted; *OR* odd ratios; *CI* confidence interval; *FDR* false discovery rate

The study found evidence of a causal link between 2 inflammatory proteins and an increased risk of developing CKD (Table. S6). The IVW method for genetic prediction revealed that higher levels of interleukin-17C (IL-17A) (OR = 1.11, 95% CI = 1.06–1.16, *p* < 0.001, FDR = 0.001) and leukemia inhibitory factor receptor (LIF-R) (OR = 1.06, 95% CI = 1.02–1.10, *p* = 0.005, FDR = 0.043) were associated with an increased risk of CKD, and the results were similar with the MR-Egger and weighted median analyses. The IVW analysis also revealed that lower levels of fibroblast growth factor 5(FGF-5) and CD40L receptor(CD40) were associated with a higher risk of CKD [(OR = 0.91, 95% CI = 0.88–0.95, *p* < 0.001, FDR = 0.001), (OR = 0.95, 95% CI = 0.93–0.98, *p* < 0.001, FDR = 0.004)], which was observed in the weighted median analyses that were consistent (*p* = 0.015, *p* = 0.004) but not in MR-Egger (*p* = 0.073, *p* = 0.080). Our analysis revealed a homogeneous effect across instrumental variables, as indicated by nonsignificant Q-statistics. Furthermore, the MR-PRESSO tests did not suggest the presence of horizontal pleiotropy (*p* > 0.05). Subsequently, a reverse-direction analysis was performed to explore the genetic associations between chronic kidney disease and the inflammatory proteins under investigation (Table S7). The IVW analysis revealed that CKD may lead to higher levels of cystatin D (CST5) (OR = 1.16, 95% CI = 1.09–1.24, *p* < 0.001, FDR = 0.001), with the results supported by the MR-Egger and weighted median analyses. And CKD may lead to elevated levels of macrophage colony-stimulating factor 1 (CSF-1) (OR = 1.13, 95% CI = 1.06–1.21, *p* < 0.001, FDR = 0.005), interleukin-15 receptor subunit alpha (IL-13) (OR = 1.13, 95% CI = 1.05–1.22, *p* = 0.001, FDR = 0.007), and programmed cell death 1 ligand 1 (PD-L1) (OR = 1.13, 95% CI = 1.06–1.21, *p* < 0.001, FDR = 0.005), with the results supported by weighted median (p < 0.05). Our study also suggests that there may be some potential causal relationship between CKD and C–C motif chemokine 23 (CCL23) (OR = 1.12, 95% CI = 1.05–1.20, *p* = 0.001, FDR = 0.007), neurotrophin-3(NT-3) (OR = 0.90, 95% CI = 0.85–0.97, *p* = 0.003, FDR = 0.018), interleukin-12 subunit beta (IL10RB) (OR = 1.12, 95% CI = 1.05–1.20, *p* = 0.001, FDR = 0.007), and vascular endothelial growth factor A(VEGF_A) (OR = 1.11, 95% CI = 1.04–1.19, *p* = 0.001, FDR = 0.012). However, the weighted median and MR-Egger estimators provided estimates of the different magnitude as the IVW analysis (*p* > 0.05). Figures 4 and 5 delineate a reciprocal causal nexus between inflammatory mediators and CKD, as established by our analyses. Scatter plot visualized the genetic associations between putative inflammatory regulators and CKD (Fig. S4). Consistency in our findings is bolstered by leave-one-out validation, which did not pinpoint any single variant with a disproportionate impact on the results (Fig. S5). Symmetry in the funnel plot further substantiates the balance and robustness of the observed associations (Fig. S6).

## Discussion

The relationship between immunological responses and chronic kidney disease has been frequently suggested by epidemiological and genetic research. Nonetheless, the intrinsic limitations of conventional epidemiology, such as uncontrolled confounding and the specter of reverse causation, preclude the establishment of definitive causal inferences from observational data alone [24]. Complicating matters further, the functional elucidation of GWAS-identified noncoding variants, predominantly situated in gene regulatory regions, poses a significant challenge. In contrast, Mendelian randomization leverages genetic variants as instrumental variables to infer causal relationships, circumventing many traditional confounders [25]. Our study pioneers a large-scale MR analysis to dissect the genetic causal associations between immune cell subtypes, inflammatory proteins, and CKD. Utilizing integrated GWAS datasets from extensive cohorts, we have discerned four immune cell subtypes and three inflammatory proteins genetically linked to CKD.

In this two-sample MR framework, we scrutinized 731 immune cell subtypes and 91 inflammatory proteins as potential causal exposures influencing CKD. Our findings implicate subtypes such as the absolute and relative counts of CD28 + CD45RA + CD8dim T cells, CD28 expression on CD39 + CD8 + T cells, and CD16 expression on CD14–CD16 + monocytes, alongside proteins including IL-17A and LIF-R, as putative precursors to CKD. Conversely, when CKD is modeled as the exposure, our data suggest a causal elevation in CST5 levels. Notably, our analyses did not unveil any evidence of reverse causation between any single biomarker and CKD, reinforcing the directionality and potential for targeted intervention within this nexus.

CD28, a costimulatory molecule integral to T cell activation, epitomizes a type I transmembrane glycoprotein within the immunoglobulin superfamily. Expressed on approximately half of human CD8 + T cells, the engagement of CD28 with its cognate ligands, CD80 and CD86, is requisite for the sustenance and full activation of T cells. This interaction augments the synthesis of interleukins, notably IL-6, and is instrumental in promoting T cell proliferation while circumventing anergy, a state of T cell nonresponsiveness [26]. The CD45RA molecule, characterized as the elongated isoform of CD45, is a hallmark of naive T cells, denoting a quiescent state prior to antigen encounter. Intriguingly, the effector memory T cells subset, TEMRA, may re-exhibit CD45RA expression upon antigen stimulation, a phenomenon associated with terminal differentiation within the CD8 + T cell lineage [27, 28]. Within the intricate milieu of the immune system, CD8 + T cells stand as sentinels, crucial for immune surveillance and host defense against pathogens and neoplastic cells. Among these, CD8dim T cells emerge as a unique subset, distinguished by a subdued expression of the CD8 marker relative to their CD8bright counterparts. This subset exhibits a distinctive cytokine profile and transcription factor expression, resonant with CD4 + T cell subsets, playing a pivotal role in modulating immune dynamics, including inflammatory responses and the orchestration of intercellular communication [29]. Our investigation delineates a causal nexus between the elevation of both absolute and relative counts of CD28 + CD45RA + CD8 + T cells and the incidence of CKD. It is paramount to recognize that the CD28 + CD45RA + CD8 + T cell cohort potentially encompasses both naive T cells, inherently expressing CD45RA and CD28, as well as TEMRA cells that have undergone re-expression of CD45RA subsequent to prior antigenic activation and differentiation.

Emerging evidence implicates the thymic production of naive T cells as a pivotal factor in CKD pathophysiology. A recent study by Iio et al. posits that diminished thymic output, marked by scant recent thymic emigrants (RTEs), portends adverse renal outcomes in nondialysis-dependent CKD patients [30]. This association underscores the potential contributory role of impaired naive T cell generation in CKD progression. Complementarily, research by Xiang et al. illustrates a direct correlation between elevated levels of highly sensitive C-reactive protein and a depletion of naive T cells in hemodialysis patients [31]. Further, investigations by Yoon et al. reveal a positive correlation between the decline in naive T cells and the exacerbation of clinical parameters such as azotemia, oxidative stress, and hyperphosphatemia in end-stage renal disease, hinting at a link with T cell senescence and subsequent renal function deterioration [32]. Moreover, naive T cells are increasingly recognized for their role in the etiology of various renal pathologies, including autoimmune glomerulonephritis. In this context, T cells, encompassing naive subsets, are identified as central to the autoimmune cascade, instigating renal injury either through peripheral activation by autoantigens and ensuing inflammatory cytokine release or through direct renal involvement and local proliferation. The intricate interplay between naive T cells and the renal microenvironment emerges as a critical element in disease onset and progression, as delineated by Suárez-Fueyo et al. [33]. The collective body of work underscores a complex interdependence between naive T cell dynamics and renal pathology, offering novel insights into CKD and autoimmune renal disorders.

Research within the CKD patient population has illuminated a decline in naive T cells and a concomitant rise in activated, terminally differentiated memory subsets, notably CD8 + TEMRA cells [34]. These TEMRA cells, whose prevalence intensifies with age, exhibit a phenotypic shift characterized by diminished TCR signaling and an upregulation of NK receptors [35], signaling a potential pivot toward an innate-like immune response. Such a transformation may impair adaptive T cell functions, including proliferation and cytokine production, thereby exacerbating CKD progression through heightened susceptibility to infections and other immunological challenges [36]. Rituximab, a monoclonal antibody targeting B cells, is noted for its capacity to modify T- and B-lymphocyte populations in steroid-dependent nephrotic syndrome (SDNS), potentially contributing to its therapeutic efficacy and the maintenance of remission [37]. Observations from a clinical trial indicated that frequencies and counts of the TEMRA subset remain unchanged up to 24 months post-transplant, irrespective of rituximab administration [38]. This stability is mirrored in kidney transplant recipients, where TEMRA CD8 + T cells were observed to sustain elevated levels one-year post-transplantation [39]. In the context of kidney transplantation, TEMRA CD8 + T cells demonstrate superior migratory capabilities in response to chemokines like CXCL12, mediated through the P2X4 receptor, underscoring their relevance as potential targets for mitigating transplant rejection [40]. In sum, the proinflammatory and cytotoxic nature of CD8 + TEMRA cells [41] implicates them as active participants in the pathogenesis and progression of CKD, highlighting their significance as subjects for further study and potential intervention within this complex clinical landscape.

CD39 expression on CD8 + T cells may confer a regulatory function analogous to that of classical regulatory T cells, underscoring its critical role in immune modulation and inflammation control. As a pivotal component in purinergic signaling, CD39 mitigates inflammation by degrading extracellular ATP, a danger signal, into adenosine, thus promoting an anti-inflammatory milieu. This enzymatic activity is crucial in maintaining the delicate equilibrium between pro-inflammatory and anti-inflammatory signals, a balance of particular relevance in chronic inflammatory states such as CKD [42, 43]. In the milieu of end-stage kidney disease, an advanced manifestation of CKD, elevated serum IFN-γ, and TNF-α levels are noted [44]. IFN-γ's extensive influence on immune responses designates it as a potential therapeutic target in hyperinflammatory diseases, including CKD [45]. Experimental findings in macrophage-specific TNF-α knockouts demonstrate attenuated renal injury and fibrosis, underscoring the therapeutic promise of TNF-α modulation in CKD, where chronic inflammation is a known driver of pathology [46].

In patients with clear cell renal cell carcinoma, CD39 + CD8 + T cells exhibited a dampened production of pro-inflammatory cytokines TNF-α and IFN-γ alongside increased expression of inhibitory markers PD-1 and TIM-3 [47]. This subtype suggests a potential for these cells to mitigate inflammatory processes within the renal environment. Complementarily, CD28's presence on these cells may signal an aptitude for robust effector functions upon activation. Our observations align with this notion, revealing an inverse causal relationship between the abundance of CD28-expressing CD39 + CD8 + T cells and the incidence and progression of CKD, implicating these cells in protective mechanisms against CKD pathogenesis.

Nonclassical monocytes, delineated by the CD14- CD16 + subtype and often termed 'patrolling' monocytes, have been implicated in the exacerbation of renal pathology. Their proclivity for inflammation and tissue damage response positions them as critical actors in disease progression, homing to sites of affliction [48, 49]. These monocytes' propensity for producing pro-inflammatory cytokines situates them at the forefront of initiating and perpetuating inflammatory cascades, particularly within the context of renal disorders such as glomerulonephritis or CKD [50]. In systemic lupus erythematosus, both murine models and clinical observations have highlighted the glomerular accumulation of these patrolling monocytes. Intravascular activation of these cells via toll-like receptor pathways has been identified as a significant contributor to glomerular inflammation and subsequent renal injury [51]. Complementarily, IgA nephropathy has been associated with an augmented presence of nonclassical monocytes relative to healthy individuals, further underscoring their pathological significance [52]. Collectively, these findings bolster the hypothesis that CD14–CD16 + monocytes play an instrumental role in fostering the inflammatory milieu that predicates renal fibrosis and deterioration. The expression level of CD16 on these monocytes emerges as a potential biomarker for the severity and progression of renal diseases, offering a window into the inflammatory undercurrents that characterize these pathologies.

Chronic kidney disease is frequently accompanied by a persistent, low-grade inflammatory state, implicated in disease progression and linked to a spectrum of comorbid conditions including atherosclerosis, cardiovascular diseases, cachexia, malnutrition, and anemia [53]. At the core of this inflammatory milieu is IL-17A, a cornerstone cytokine within the IL-17 family, renowned for its proinflammatory actions in synergy with cytokines like IL-1-β and TNF. These interactions, however, exhibit distinct mechanistic pathways contingent upon cellular context and disease states [54, 55]. Elevated IL-17A levels have been associated with nephrotic hypertension in CKD patients [56] and are posited as a predictor for nonalbuminuric CKD [57]. Recent paradigms propose IL-17A as a therapeutic target in CKD, with evidence suggesting that IL-17A inhibition ameliorates damage from peritoneal dialysis fluids [58]. The deleterious role of IL-17A in renal pathology is further exemplified by Th17 cell delivery in mice, precipitating albuminuria, glomerular neutrophilic infiltration, and renal CXCL1 mRNA elevation [59]. Leukemia inhibitory factor receptor (LIF-R), a receptor for the multifunctional cytokines like LIF, is intimately connected with renal interstitial fibrosis and CKD progression. LIF, part of the IL-6 cytokine family, is notably upregulated in fibrotic renal lesions, inversely correlating with eGFR, and presenting as a potential biomarker for CKD [60]. LIF-R's modulation has shown promise in experimental models, where its knockdown mitigates RIF, while its upregulation aggravates it, marking the LIF/LIF-R axis as a potential therapeutic target for kidney fibrosis and CKD progression. Furthermore, LIFR gene anomalies have been detected in a subset of patients with congenital anomalies of the kidney and urinary tract [61]. Our findings corroborate the causal relationship between elevated IL-17A, LIF-R expression, and CKD, enriching the understanding of CKD's inflammatory underpinnings and offering novel avenues for targeted therapy.

Cystatin D, encoded by CST5, is a member of the cystatin superfamily, which also includes cystatin C, encoded by CST3. Northern blot analyses have delineated cystatin D's expression as predominantly localized to the parotid gland, exhibiting a more restricted tissue distribution compared to the ubiquitously expressed cystatin C. Despite cystatin C's established role as an early and sensitive biomarker for CKD, facilitating diagnosis especially in scenarios where creatinine is inadequate [62], the implications of cystatin D in renal pathology remain underexplored. Cystatin D has demonstrated potential as an acute-phase marker, distinguishing patients with severe traumatic brain injury within the first hour post-event, indicative of its involvement in early inflammatory responses [63]. It has been identified as a p53 target [64] and exhibits tumor suppressor functions in colon cancer by counteracting the Wnt/beta-catenin pathway and inhibiting cellular migration [65]. Additionally, cystatin D has been shown to inhibit osteoclast activation and resorption, modulating the NF-κB signaling pathway [66]. Furthermore, CST5 has emerged as a significant correlate of 28-day mortality post-acute myocardial infarction [67]. Recent investigations, including a comprehensive German diabetes study encompassing both type 1 and type 2 diabetic patients, have revealed an inverse correlation between CST5 levels and estimated glomerular filtration rate (eGFR), positing a potential role for cystatin D in CKD progression [68]. Our study corroborates these findings, elucidating a direct causal link between CST5 and CKD, thereby expanding the understanding of cystatin D beyond its established biological functions and into the realm of kidney disease pathogenesis.

Our study is subject to several limitations. First, our study's conclusions, proposing a causal link between CD28 + CD45RA + CD8 + T cell populations and CKD, should be interpreted in the context of inherent methodological constraints. Notably, this T cell cohort encompasses both naive T cells, characterized by CD45RA and CD28 expression, and a specialized subset of TEMRA cells that re-express CD45RA post-activation. Distinguishing these subsets definitively necessitates additional markers and functional assays, such as CCR7 expression, senescence indicators, and analyses of cytokine production and proliferative response to stimulation [69]. Second, the scope of our MR analysis is further circumscribed by the limited scale of the lymphocyte phenotyping GWAS available, which constrains the pool of variants suitable for use as IVs. Although adopting a less stringent association threshold yields more IVs, this approach could undermine the stringent MR assumption of robust association with the exposure. Prospective larger-scale phenotyping GWAS is anticipated to provide a broader array of SNPs meeting the GWAS significance threshold for more reliable MR analysis. Third, our findings are potentially subject to ethnic bias, as the cohorts predominantly comprised individuals of European descent, which may not extrapolate across different ethnic groups. Finally, while MR is an instrumental approach for causal estimation, it is not a panacea for randomized controlled trials (RCTs). Hence, the causal inferences drawn from this analysis may not fully concur with RCT outcomes. Future research directions should integrate individual-based genetic investigations and RCTs to substantiate the causative associations proposed herein.

## Conclusion

Our study represents a significant advancement in the elucidation of the genetic interplay underpinning CKD, employing Mendelian randomization to illuminate the causal pathways between immune cell subtypes, inflammatory mediators, and CKD. By leveraging robust GWAS datasets, we have identified several immune markers, including specific T cell populations and inflammatory proteins such as IL-17A and LIF-R, that may precipitate the onset and progression of CKD. Conversely, CKD itself appears to instigate alterations in CST5 levels, revealing a bidirectional relationship between renal dysfunction and immune responses. Future research is imperative to substantiate these genetic correlations and to integrate them into a precision medicine framework, with the ultimate goal of devising targeted therapeutic strategies for CKD.

## Supplementary Information

Below is the link to the electronic supplementary material.

**Figure S1** Leave-one-out plots for immune cells on CKD Supplementary file1 (TIF 2736 kb)

**Figure S2** Funnel plots for immune cells on CKD Supplementary file2 (TIF 1974 kb)

**Figure S3** Scatter plots for immune cells on CKD Supplementary file3 (TIF 2849 kb)

**Figure S4** Scatter plots for inflammation proteins on CKD Supplementary file4 (TIF 2228 kb)

**Figure S5** Leave-one-out plots for inflammation proteins on CKD Supplementary file5 (TIF 2125 kb)

**Figure S6** Funnel plots for inflammation proteins on CKD Supplementary file6 (TIF 1595 kb)

**Table S1** Immune cell traits information Supplementary file7 (CSV 28 kb)

**Table S2** Inflammation traits information. Supplementary file8 (CSV 18 kb)

**Table S3** The characteristics of SNPs for CKD Supplementary file9 (XLSX 124 kb)

**Table S4** Forward MR analyses of causal associations between immune cells and CKD Supplementary file10 (CSV 492 kb)

**Table S5** Reverse MR analyses of causal associations between CKD and immune cells Supplementary file11 (CSV 3 kb)

**Table S6** Forward MR analyses of causal associations between inflammation proteins and CKD Supplementary file12 (CSV 56 kb)

**Table S7** Reverse MR analyses of causal associations between CKD and inflammation proteins Supplementary file13 (CSV 54 kb)

## Data Availability

Publicly available datasets were analyzed in this study. These data can be found here: https://ckdgen.imbi.uni-freiburg.de/; https://www.ebi.ac.uk/gwas/home. All the data generated by the MR analysis are in the included Supplementary Material.

## Abbreviations

CKD: Chronic kidney disease
GWAS: Genome-wide association study
ESRD: End-stage renal disease
MR: Mendelian randomization
IVs: Instrumental variables
LD: Linkage disequilibrium
IVW: Inverse variance-weighted
WM: Weighted median
InSIDE: Instrument Strength Independent of Direct Effect
MR-PRESSO: MR pleiotropy residual sum and outlier
LOO: Leave-one-out
OR: Odds ratio
CI: Confidence interval
FDR: False discovery rate
MFIs: Median fluorescence intensities
RTEs: Recent thymic emigrants
SDNS: Steroid-dependent nephrotic syndrome
eGFR: Estimated glomerular filtration rate
