# Supplementary figures and images for: Immune cell signatures and inflammatory mediators: unraveling their genetic impact on chronic kidney disease through Mendelian randomization
Source: Clin Exp Med. 2024 May 4;24(1):94. doi: 10.1007/s10238-024-01341-z (PMC11069478; doi:10.1007/s10238-024-01341-z)

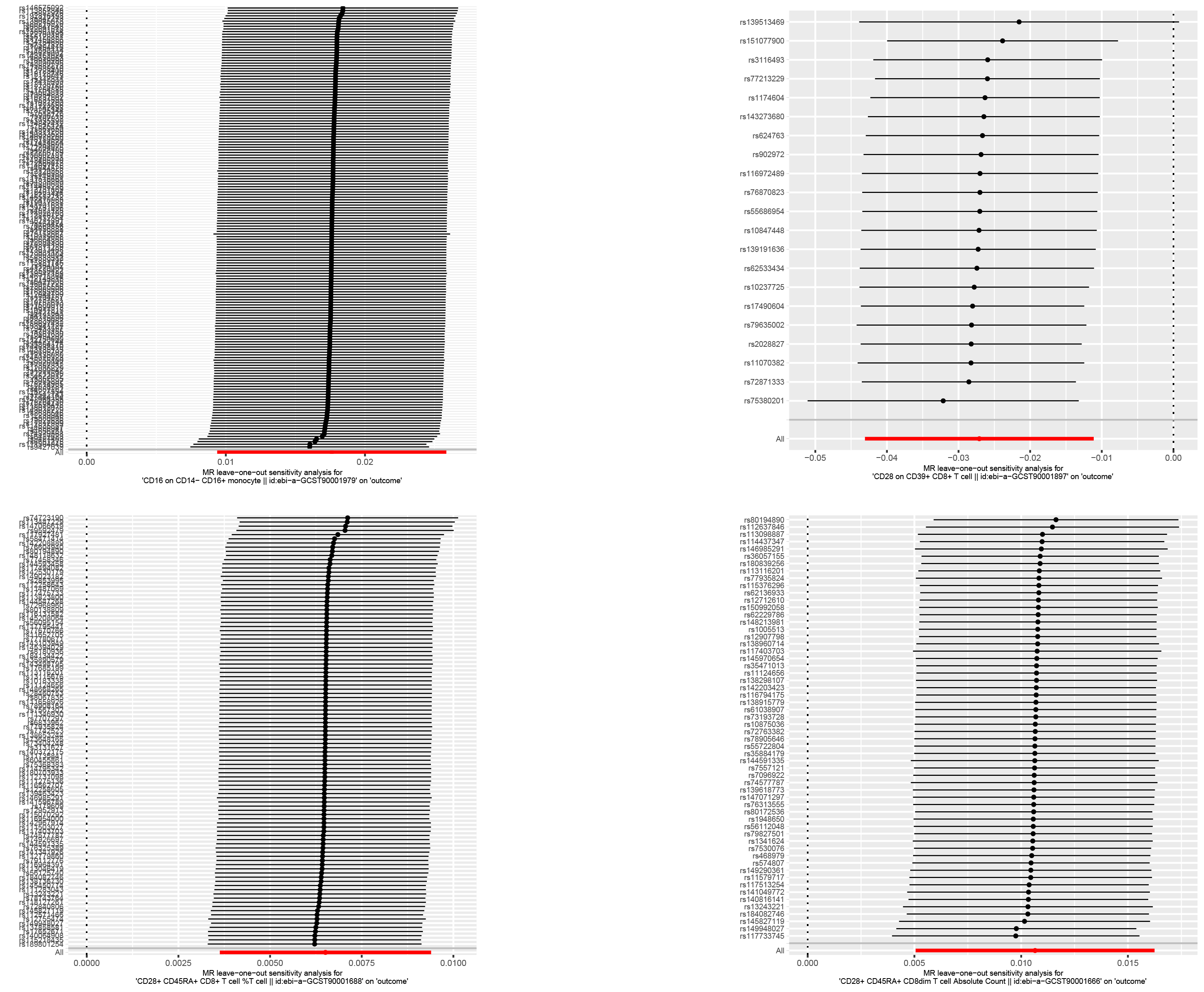

Supplement: Supplementary file 1 — Figure S1 Leave-one-out plots for immune cells on CKD Supplementary file1 (TIF 2736 kb) [file 10238_2024_1341_MOESM1_ESM.tif]

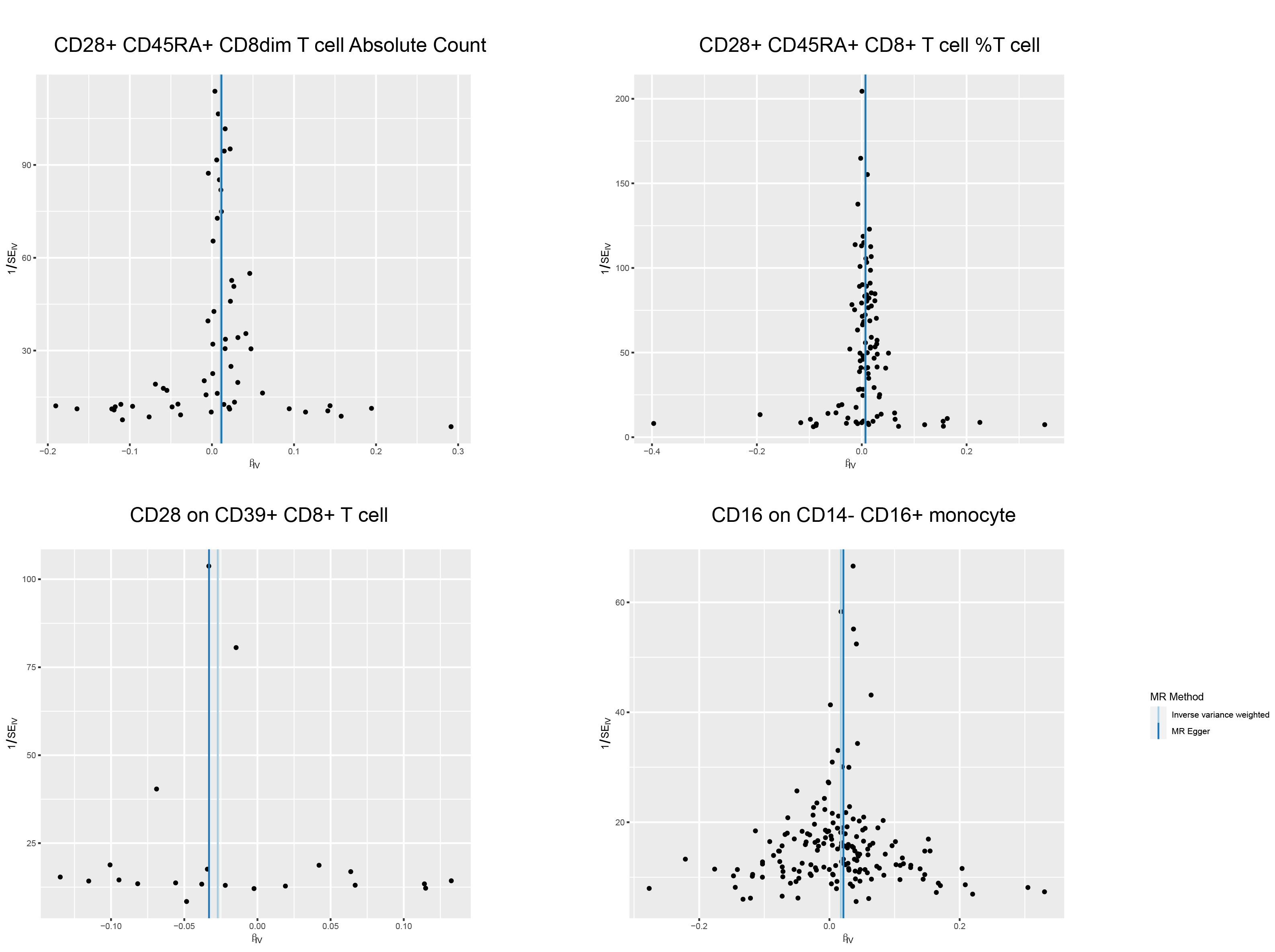

Supplement: Supplementary file 2 — Figure S2 Funnel plots for immune cells on CKD Supplementary file2 (TIF 1974 kb) [file 10238_2024_1341_MOESM2_ESM.tif]

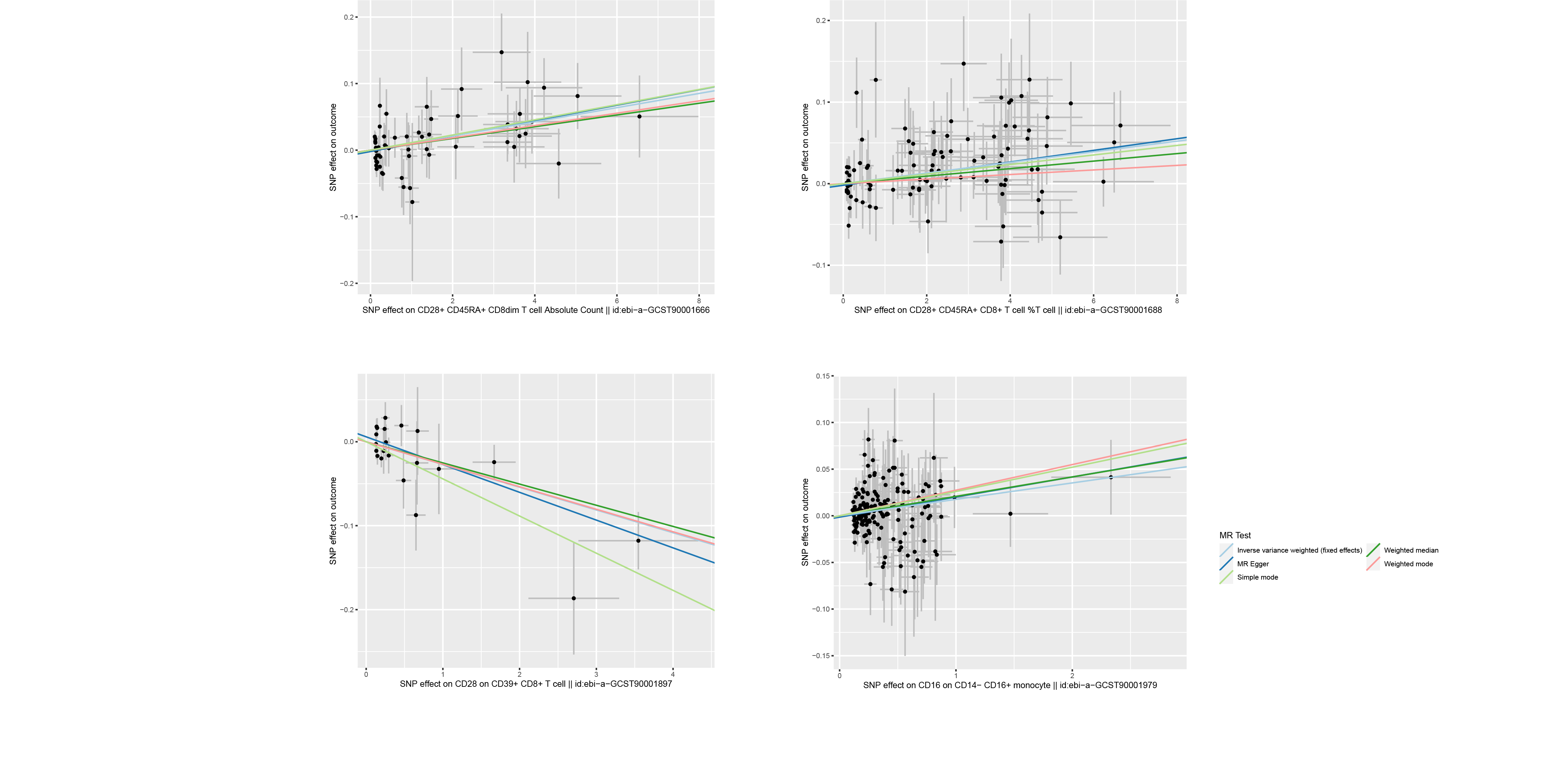

Supplement: Supplementary file 3 — Figure S3 Scatter plots for immune cells on CKD Supplementary file3 (TIF 2849 kb) [file 10238_2024_1341_MOESM3_ESM.tif]

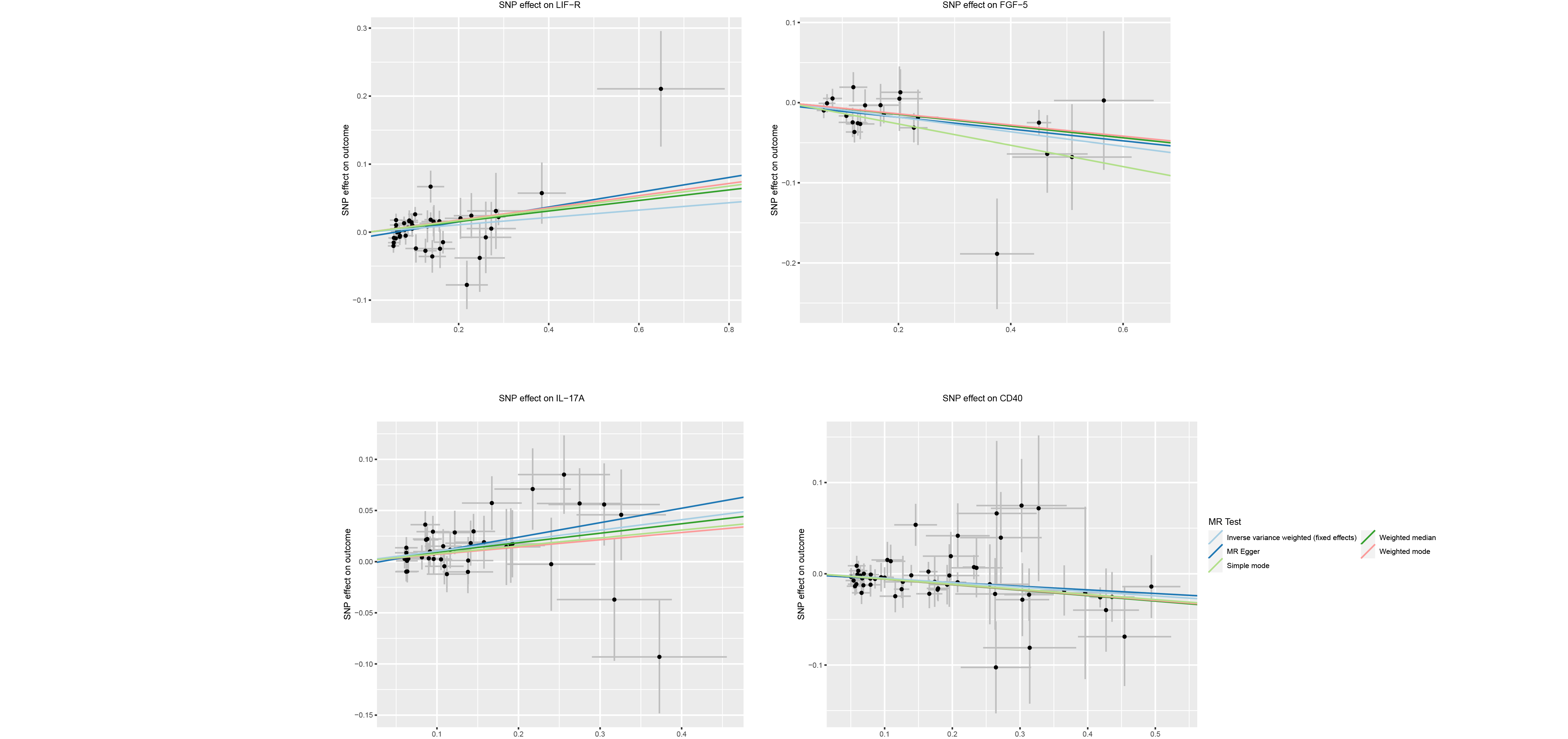

Supplement: Supplementary file 4 — Figure S4 Scatter plots for inflammation proteins on CKD Supplementary file4 (TIF 2228 kb) [file 10238_2024_1341_MOESM4_ESM.tif]

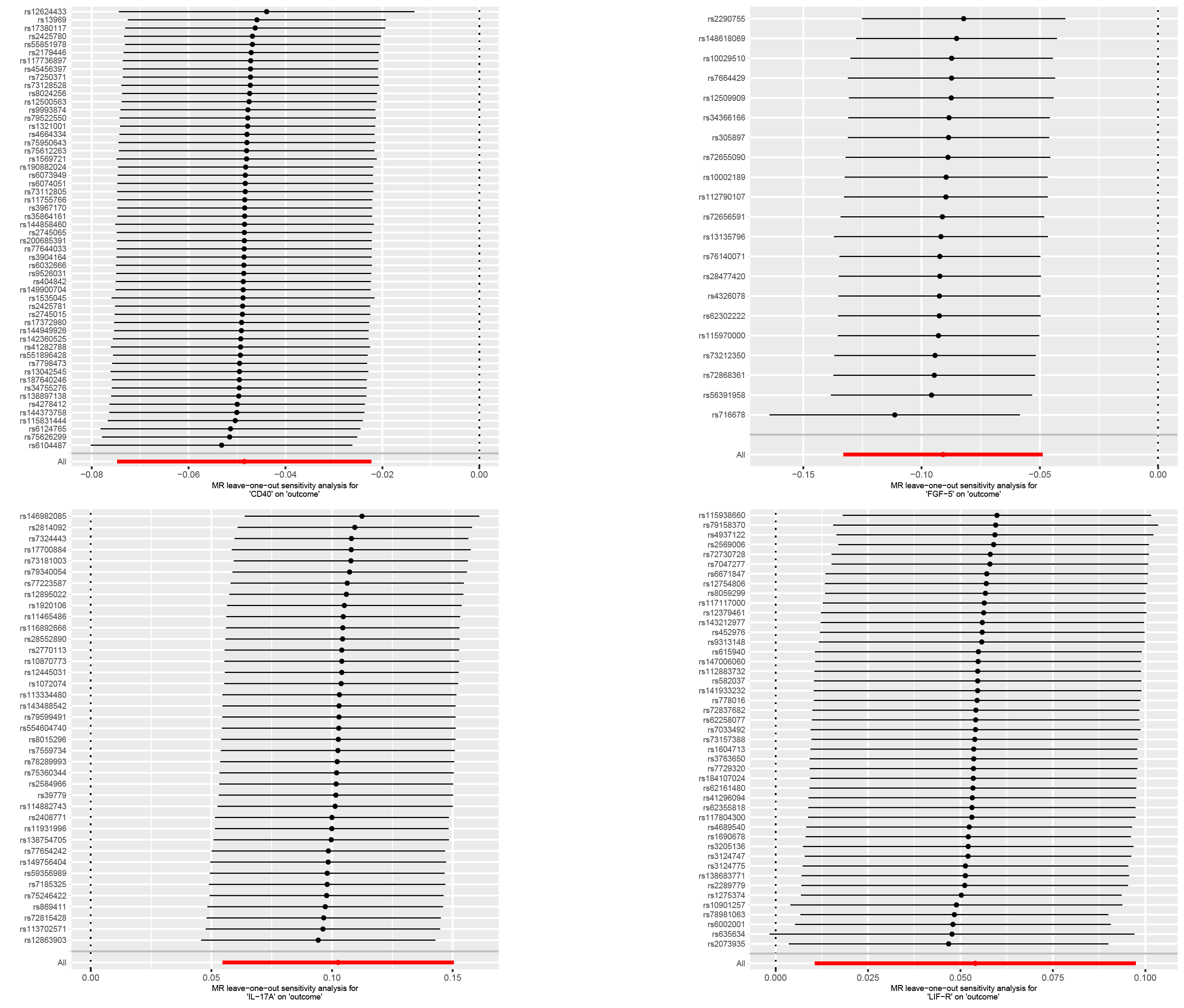

Supplement: Supplementary file 5 — Figure S5 Leave-one-out plots for inflammation proteins on CKD Supplementary file5 (TIF 2125 kb) [file 10238_2024_1341_MOESM5_ESM.tif]

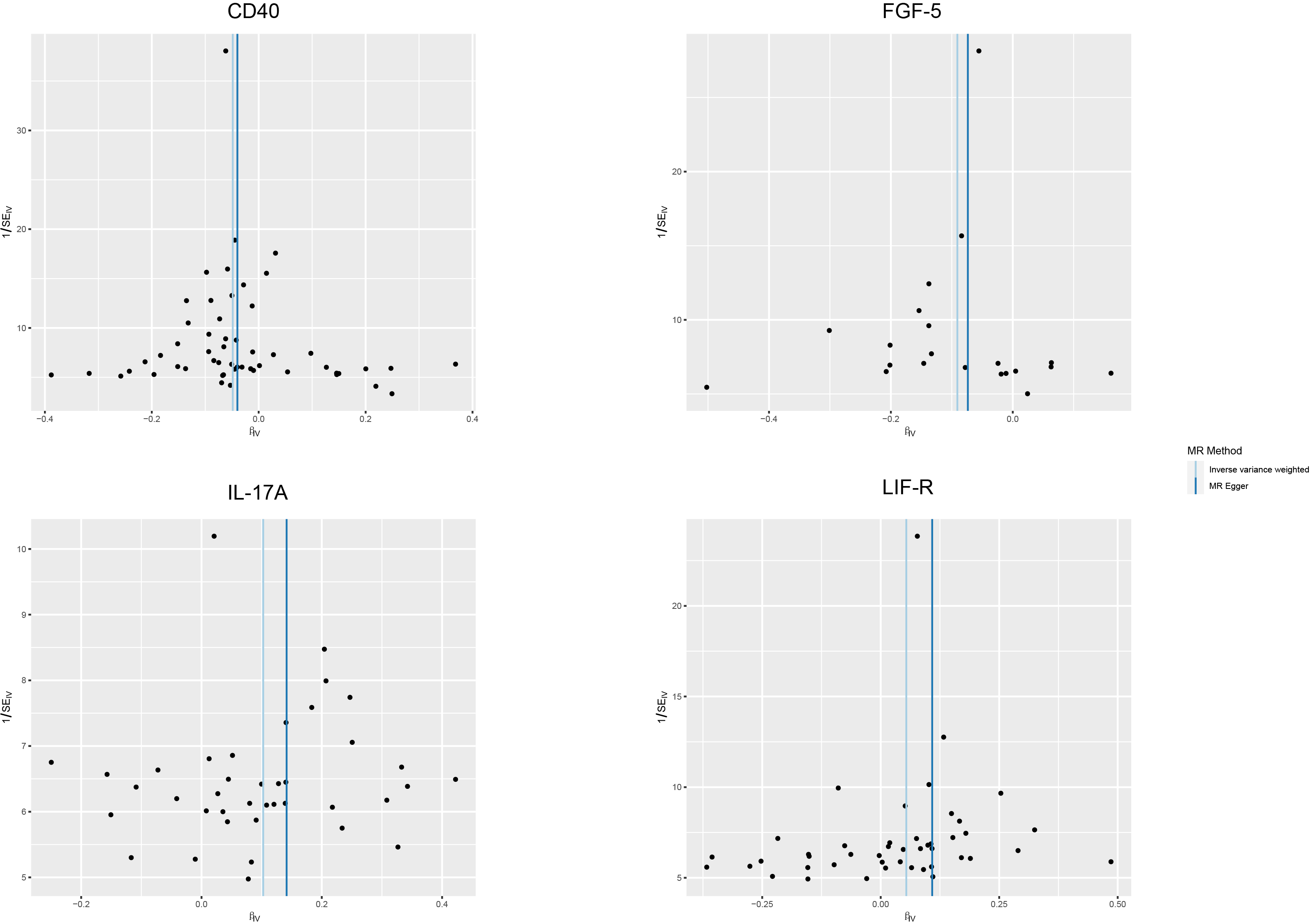

Supplement: Supplementary file 6 — Figure S6 Funnel plots for inflammation proteins on CKD Supplementary file6 (TIF 1595 kb) [file 10238_2024_1341_MOESM6_ESM.tif]
